# AMPICON 2006: A view point

**DOI:** 10.4103/0971-6203.28016

**Published:** 2006

**Authors:** G. S. Pant

**Affiliations:** Chairman, Scientific Program Committee, AMPICON 2006, Professor (Med Phys), Dept. of Nuclear Medicine, AIIMS, Convener Scientific Program AMPICON 2006, New Delhi, India

The Association of Medical Physicists of India (AMPI), now 30 years young, was founded in the year 1976 with the objective of promoting the application of physics in medicine and biology. It provides a forum for medical physicists in the form of annual conferences (at national and international levels) and publication of a quarterly Journal of Medical Physics for the exchange of ideas and dissemination of knowledge. The host of the AMPI Conference is decided by the general body of the association on receipt of the formal request from the head of the host institution. The association and the host institution are jointly organizing the conference.

The 27^th^ annual conference of the association is being organised at Bhubaneswar, Orissa during 9-12 November 2006. This is also the seventh International Conference on Medical Physics (ICMP) in India. The host of this conference, the Hemalata Hospitals and Research Centre, Bhubaneswar is equipped with all the adequate facilities for the treatment of cancer. It is pleasure to know that this hospital has come to this stage by the relentless efforts made by one of our medical physics colleagues, Dr. A.K. Rath who is also the Chairman of the organising committee of the conference.

This issue of the journal is exclusively dedicated to AMPICON 2006. About 200 abstracts were received by the organisers, which were evaluated by the scientific committee for presentation at the conference. Extended abstracts of proffered papers -both oral (63) and poster (127) - are included in this issue. The scientific programme consists of invited talks including Ramaiah Naidu Memorial oration. Ramaiah Naidu Memorial Oration (RNMO) Award was instituted by the association in 1992 in the honour of pioneer Indian medical physicist Dr. Ramaiah Naidu. The oration award consists of a citation and a silver plaque. As per the norms of AMPI, the recipient of RNMO award shall be a person of national/ international repute in the field of medical physics. The recipient of this award is selected by the Executive Committee of AMPI. There are 14 scientific sessions, including 19 invited talks, 2 guest lectures and 3 poster sessions. For the first time the scientific programme includes a debate on a topic that I am sure that all the medical physicists in radiation oncology will find it interesting. Keeping in line with the recent trends in radiation oncology three invited talks are being organised on image guided radiotherapy (IGRT) by experts from India and abroad. It is also decided to keep early morning lectures on various topics for the benefit of our young colleagues in the specialty.

While going through the abstracts, the scientific committee felt that there is scope for improvements in the methodology for abstract submission, their speedy evaluation and informing the authors on decision taken. We will certainly pass on our suggestions to the association for consideration. Couple of years down the line I hope that complete process of submission to publication of abstracts should be made on-line.

As a guest editor I take this opportunity to request all the authors of proffered papers to prepare the full manuscript of their good work and submit to the editorial office for publication in this journal.

I also thank the members of scientific and publication committees for their full cooperation and hard work in bringing out this issue in time.

